# Characterization of the Age-Dependent Changes in Antioxidant Defenses and Protein’s Sulfhydryl/Carbonyl Stress in Human Follicular Fluid

**DOI:** 10.3390/antiox9100927

**Published:** 2020-09-28

**Authors:** Alice Luddi, Laura Governini, Angela Capaldo, Giovanna Campanella, Vincenzo De Leo, Paola Piomboni, Giuseppe Morgante

**Affiliations:** Department of Molecular and Developmental Medicine, University of Siena, 53100 Siena, Italy; luddi@unisi.it (A.L.); laura.governini@unisi.it (L.G.); angela.ca@live.it (A.C.); giovanna.x.campanella@gmail.com (G.C.); vincenzo.deleo@unisi.it (V.D.L.); giuseppe.morgante@unisi.it (G.M.)

**Keywords:** human follicular fluid, aging, oxidative stress, reactive oxygen species (ROS)

## Abstract

The oxidative stress, characterized by the imbalance between pro-oxidants and antioxidants molecules, seems to be involved in the pathogenesis of female subfertility. In particular, the presence of different markers of oxidative stress has been reported in human follicular fluid (FF) surrounding oocytes. Based on its distinctive composition and on the close proximity to the oocyte, FF creates a unique microenvironment having a direct impact on oocyte quality, implantation, and early embryo development. An imbalance in reactive oxygen species (ROS) production in ovarian follicular fluid may have a negative effect on these processes and, as a consequence, on female fertility. Therefore, the aim of this study was to evaluate the redox state of the FF through various methodological approaches. By means of 2D-electrophoresis we demonstrated that the main structural changes occurring in the proteins of the follicular fluid of normovulatory women were correlated to the age of the patients and to the antioxidant defenses present in the FF. Measurement of these parameters could have clinical relevance, since the assessment of the oxidative stress rate may be helpful in evaluating in vitro fertilization potential.

## 1. Introduction

The ovarian follicle, the natural milieu where the oocyte growths, is the metabolically active beating heart where a complex network of signalling pathways guarantees the appropriate oocyte maturation, which is needed for fertilization to take place [1]. In this context, follicular fluid (FF) represents an important component of the oocyte microenvironment significantly contributing to the appropriate follicular growth and oocytes maturation. FF is a product of both the diffusion of blood molecules through the blood follicular barrier and of the secretory activity of ovarian somatic cells (granulosa and theca cells) and oocyte [2,3]. Its biochemical composition reflects the functional state of the follicle, and, as a consequence, the oocyte competence, which in turn affects oocyte quality and its potential to achieve fertilization [4,5]. Noteworthy, FF represents an effective option for the indirect evaluation of oocyte quality since it is an abundant biological sample that can be easily collected without compromising the oocyte quality during the assisted reproductive procedures. Therefore, numerous studies investigating the molecular composition of FF have led to the identification of biomarkers for a wide range of fertility issues [3].

A great deal of research pointed to the possible role of oxidative stress in follicular fluid as causative factors of female infertility [6,7,8,9]. It is known that FF contains both reactive oxygen species (ROS) and antioxidant enzymes, with ROS being physiologically produced during the ovulatory process; indeed, ROS are key molecules in regulating different phases of physiological reproductive functions, such as oocyte maturation, ovarian steroid production and corpus luteal functions, as well as in fertilization and embryo development [10,11]. Under physiological conditions, antioxidant defense systems prevent ROS production and scavenge existing free radicals; any unbalance between ROS production and the antioxidant defense systems results in an increase of ROS and induces an oxidative stress (OS) status that, in turn, may directly affect ovarian somatic cells and oocyte physiology. A huge amount of data support that ROS significantly speed up ovarian aging, a pathological condition characterized by the gradual qualitative and quantitative worsening of the ovarian oocyte reserve, resulting in decreased oocyte competence together with reduced natural fertility [12,13]. In this regard, it has been proven that in mice, an antioxidant supplementation is able to counteract the detrimental effects of aging on oocyte quantity and quality [14].

ROS are highly reactive electrophiles able to oxidize nucleophilic functional groups (such as hydroxyl group -OH, amminic group -NH_2_, and sulfhydryl group -SH) of polysaccharides, nucleic acids, and proteins; oxidation induces harmful effects on these biomolecules that, if not promptly repaired, may lead to cell injury and death [15].

Noteworthy, proteins are the most effective target of oxidative damage because the effect of damage to one molecule is greater than stoichiometric. Moreover, since thiols may undergo reversible oxidization, -SH is more reactive than -OH and -NH_2_ and, consequently, proteins having sulphur-containing amino acids, are more susceptible to the attack of ROS [9,16,17]. On the other end, carbonyl (CO) groups (aldehydes and ketones) are produced on protein side chains when they are oxidised. Carbonylated proteins are formed early in oxidative stress conditions and are relatively stables, therefore protein CO groups may be regarded as biomarkers of oxidative stress and their measurement represents a gold standard in OS assessment [18].

On this basis, the aim of this study was to measure the non-enzymatic antioxidant defenses in the follicular fluid of women undergoing in vitro fertilization (IVF) cycles, investigating their behaviour with ageing. Moreover, we studied the direct effects of OS on proteins, through the analysis of free thiol and carbonyl groups, as specific and sensitive markers of oxidative stress.

## 2. Materials and Methods

### 2.1. Patients Enrolment and Ovulation Induction

The study, performed at the Centre for Couple Sterility, Siena University Hospital was approved by Ethic Committees of Siena University Hospital. A total of 40 normovulatory women undergoing their first IVF treatment, between January 2018 and December 2018, were enrolled after signing an informed consent. All patients didn’t achieve a pregnancy after at least one year of unprotected sexual intercourse. The reasons for the couples’ infertility were male factor infertility (*n*  =  28) or tubal occlusion (*n*  =  12). Ovarian stimulation was performed administering recombinant gonadotropins (Gonal F-Merck Serono, Italy, and Puregon, (MSD, Merck Sharp & Dohme Corp., Inc., Kenilworth, NJ, USA.) at the dose of 150–300 IU per day of from the 1nd or 2nd day of induced menstruation. According to the ovarian response, detected by serial transvaginal ultrasound examination, the dose of gonadotropins was adjusted. Transvaginal scans were performed using a Voluson 6 (GE Healthcare, Milwaukee, WI, USA) machine, equipped with a 7–10 MHz two-dimensional transvaginal probe. During ovarian stimulation, patients were evaluated every two days for ultrasound monitoring and serum routine hormonal measurement (Follicular stimulating hormone, FSH; luteinizing hormone, LH; estradiol, E2) in order to evaluate the ovarian response. When the dominant follicle reached 14 mm in diameter, a Gonadotropin Releasing Hormone GnRH antagonist (Orgalutran, MSD, Merck Sharp & Dohme Corp., Inc., Kenilworth, NJ, USA and Cetrotide, Merck Serono, Roma, Italy) was administered daily, until the day of ovulation triggering; ovulation was induced by recombinant human chorionic gonadotropin (hCG) injection (Gonasi 10,000 IU, IBSA, Lodi, Italy), when at least three follicles of size >18 mm were present in the ovaries. 34–36 h after the hCG injection, the oocyte pick-up was performed. As soon as the oocytes were collected, FF was centrifuged for 10 min at 1500 rpm and stored at −80 °C. Only FF samples with no macroscopic evidence of blood were selected.

### 2.2. Trolox Equivalent Antioxidant Capacity (TEAC) Assay

Free radical scavenging activity of FF was determined by TEAC-ABTS assay. Staring from the stock solutions, including 7 mM of 2,2′-Azinobis-(3-Ethylbenzthiazolin-6-Sulfonic Acid) (ABTS) solution and 2.4 mM potassium persulfate solution, the working solution was then prepared by mixing 1:1 (*v/v*) the two stock solutions, stored in the dark for 14 h at room temperature, before the use. For each assay, 1 mL of this solution was added to 60 mL methanol to obtain an absorbance of 0.706 ± 0.01 units at 734 nm. Follicular fluids (50 µL of a dilution 1:5 in phosphate saline buffer, PBS) were allowed to react with 1 mL of the ABTS solution and the absorbance was measured at 734 nm after 6 min. This was compared to a blank where 50 μL of the solvent was added to the ABTS solution. The ABTS scavenging capacity of the FF was calculated by relating this decrease in absorbance to that of a Trolox solution. All the determinations were performed in triplicates.

### 2.3. Ferric Reducing Antioxidant Power (FRAP) Assay

The antioxidant capacity of the follicular fluids was estimated spectrophotometrically following the procedure previously described [8]. The freshly ready FRAP solution was set up by mixing 300 mM acetate buffer, 10 mM 2,4,6-tri-(2-pyridyl)-1,3,5-triazine (prepared in 40 mM HCl) and 20 mM FeCl_3_·6H_2_O in the ratio of 10:1:1 (*v/v/v*). 50 µL of this working solution was added with 50 μL of the FF and, 10 min later, the absorbance was measured at 593 nm. The calibration curve was prepared by plotting the absorbance at 593 nm versus different concentrations of FeSO_4_. All the determinations were performed in triplicates. All measurements were taken at room temperature with samples protected from direct sunlight.

### 2.4. Colorimetric Thiol Detection of -SH Groups

In all follicular fluid samples, the thiol detection has been carried out by using 5,5′-dithiobis-(2-nitrobenzoic) acid (DTNB), also known as Ellman’s reagent. To this end, 1 mL of 0.1 M Tris and 10 mM ethylenediaminetetraacetic acid (EDTA) pH 8.2 solution has been added with 75 µL of 10 mM DTNB solution (prepared in methanol) and 75 µL of FF. After 15 min of incubation at room temperature, the absorbance was measured at 412 nm.

### 2.5. Analysis of Follicular Fluid Proteins

#### 2.5.1. Identification of Free -SH Groups by Staining with 3-*N*-maleimidopropionyl biocytin (MPB)

We carried out the SH-groups labelling according to de Lamirande and Gagnon [19], with some modifications. Briefly, protein concentration was determined by Bicinchoninic Acid Kit (Sigma-Aldrich, St. Louis, MO, USA) following the manufacturer’s instruction. Equivalent amount of FF proteins and a solution of MPB (Sigma Aldrich, St. Louis, MO, USA) 1 mM in Tris-HCl 15 mM pH 6.8 were mixed, in a final volume of 100 μL. The mixture was then heated at 95 °C for 5 min and immediately processed for Sodium dodecyl sulfate (SDS) page and for two-dimensional (2D) electrophoresis.

#### 2.5.2. Identification of Carbonyl Groups by Staining with 2,4-dinitrophenylhydrazine (DNPH)

To identify carbonyl groups, protein carbonyls were stained by using DNPH. To this end, 120 μg FF proteins were dissolved in 6% SDS, precipitated with 2d-CleanUp Kit (Merck KGaA, Darmstadt, Germany) and incubated with an equal volume of 10 mM DNPH in 10% trifluoroacetic acid, at room temperature for 15 min. This solution was then neutralized and prepared for loading onto SDS-gels by addition of 2 M Tris base/30% glycerol (*v/v*) to achieve final concentrations of 0.52 M and 7.8% (*v/v*), respectively. Samples were immediately processed for 2D electrophoresis.

#### 2.5.3. SDS-PAGE

Protein extracts were suspended in sample buffer containing 20% (*w/v*) glycerol, 240 mM Tris-HCl, pH 6.8, 8% SDS (*w/v*), 8% (*w/v*) β-mercaptoethanol and 0.02% (*w/v*) bromophenol blue, then loaded in a 6–16 % gradient of acrylamide/bisacrylamide and finally separated by electrophoresis according to our protocol previously described [9].

#### 2.5.4. Two-Dimensional Electrophoresis

2D electrophoresis was carried out as previously reported [20,21]; briefly, 30 μg of total proteins from each sample were resuspended in rehydration solution; the samples were mixed with 0.2 % immobilized pH gradient buffer (immobilized pH gradient, IPG, GE Healthcare, Uppsala, Sweden) and then loaded onto Immobiline Dry-Strips with immobilized nonlinear pH gradient, ranging from pH 3 to 11 (GE Healthcare, Uppsala, Sweden). Runs were carried out using an Ettan™ IPGphor™ system (GE Healthcare, Uppsala, Sweden) at 16 °C. Electrical conditions were as follows: 0 V for 1 h, 30 V for 8 h, 200 V for 1 h, from 300 to 3500 V in 30 min, 3500 V for 3 h, from 3500 to 8000 V in 30 min, 8000 V for a total of 80,000 Vh. Between the two dimensions, each isoelectric strip was equilibrated for 15 min in the equilibration solution (50 mM Tris-HCl pH 6.8 containing 30% glycerol, 6 M urea, and 2% sodium dodecyl sulfate) and for an additional 5 min in the same solution with 2.5% iodoacetamide (IAA) and 0.1 % bromophenol blue, and then placed on a 6–16% polyacrylamide linear gradient SDS gel according to Laemmli et al. [22]. Linear gradient gels (9–16% SDS polyacrylamide) were used for the protein separation at 40 mA·gel^−1^ constant current and 9 °C until the dye front reached the bottom of the gel.

Analytical gels were stained with ammoniacal silver nitrate and digitalized using an Image Scanner III laser densitometer (LabScan 6.0 software; GE Healthcare, Uppsala, Sweden). Gel images were digitized with a Molecular Dynamics 300 S laser densitometer (4000 × 5000 pixels, 12 bits/pixel; Sunnyvale, CA, USA).

#### 2.5.5. Western Blotting

After electrophoresis, proteins were transferred to nitrocellulose membrane [23], that were then blocked for 1 h in 50 mM Tris-HCl, 154 mM NaCl, 0.1% Tween-20, pH 7.5 with 5% non-fat dry milk. For free SH-groups staining, the membranes were then incubated overnight with avidin in tris-buffered saline (TBS) containing 0.2% Tween 20, with 1% non-fat dry milk, washed 2 times in tween-TBS (TTBS) and then incubated for 1 h with streptavidin conjugated to peroxidase. For the carbonyl-groups staining, membranes were incubated overnight at 4 °C with a rabbit anti-DNP antibody (1:150; Chemicon, Merck-Millipore, Massachusetts, USA). The secondary antibody horseradish peroxidase (HRP)-conjugated anti-rabbit IgG (1:300; Chemicon, Merck-Millipore, Massachusetts, USA) was incubated for 1 h at room temperature. A positive and a negative control was used to determine the staining specificity. Immuno-Star HRP Chemiluminescent kit (Bio-Rad Microsciences, Hemel Hempstead, UK) was used to detect the immunoreactivity then revealed with an XRS instrument ChemiDoc (Bio-Rad Microsciences, Hemel Hempstead, UK). Spots identification and quantification as pixel/mm2 were performed by using the Quantity One^®^ 4.5.7 and PDQuest^TM^ 7.4.0 softwares (Bio-Rad Microsciences, city, country). For the carbonyl staining, Oxy-Blot^TM^ Protein Oxidation Detection Kit (Chemicon, Merck-Millipore, Massachusetts, USA) was used

### 2.6. Isolation and Identification of Proteins of Interest by MALDI-TOF (Matrix-Assisted Laser Desorption/Ionization Time Of Flight)

After silver staining compatible with mass spectrometry, the preparative gel was matched to the master gel and a spot-picking list was generated. Protein spots were mechanically excised from at least two different 2-D preparative gels by an Ettan Spot Picker (GE Healthcare, Uppsala, Sweden). Reference gels summarizing all spot identifications were defined. The spots, mechanically excised, were destained in 2.5 mM ammonium bicarbonate and 50% acetonitrile, dehydrated in acetonitrile and then rehydrated in a trypsin solution overnight at 37 °C; 0.75 µL of each digested protein were spotted into the MALDI target and dried. After that, 0.75 mL of the matrix solution (saturated solution of α-cyano-4-hydroxycinnamic acid in 50% acetonitrile and 0.5% *v/v* trifluoroacetic acid) was added to samples and then dried. A mass fingerprinting search was carried out in Swiss-Prot/TrEMBL and NCBInr databases using the online-available software MASCOT (version 2.6, Matrix Science Ltd., London, UK, http://www.matrixscience.com; threshold 50). Taxonomy was limited to *Homo sapiens*, a mass tolerance of 100 ppm was allowed and the number of accepted missed cleavage sites was set to one. Alkylation of cysteine by carbamidomethylation was considered as a fixed modification, while oxidation of methionine was considered as a variable modification. The criteria used to accept identifications included the extent of sequence coverage, the number of matched peptides and the MASCOT algorithm assigned probabilistic score (*p* < 0.05; score > 60).

### 2.7. Image and Statistical Analysis

ImageMaster 2D Platinum v7.0 software (GE Healthcare, Uppsala, Sweden) was used to analyze each image of gels. The reference gel was defined and used for the comparative analyses. Statistical analysis for protein differently expressed in the groups was carried out using GraphPad Prism software and the MedCalc version 12.1.4 statistical software package (MedCalc Software, Mariakerke, Belgium) was used. We considered “differently expressed” those spots being unmatched or having a significantly different percentage volume (%V) Nonparametric Mann–Whitney rank sum test or Kruskal–Wallis analysis of variance were used, as appropriate, to test the differences between groups. A two-sided *p* < 0.05 was considered to indicate statistical significance.

### 2.8. Enrichment Analysis by Search Tool for the Retrieval of Interacting Genes/Proteins (STRING) Software

In order to perform STRING analysis, the list of the UniProt accession numbers of the identified proteins was loaded in STRING software by “multiple protein” option. *Homo sapiens* was selected as preferred “organism”. In the “settings” section, “molecular action” was selected as the preferred interaction among proteins to be visualized. Textmining, Experiments, Databases and co-expression were selected as active interaction sources. The minimum required interaction score was 0.700 considered of high confidence. No more than five interactors were permitted to be added. The network view summarizes the predicted associations for a particular group of proteins. The nodes represent proteins, while the edges represent the predicted functional associations. Nodes are modelled as masses and edges as springs. For this reason, the final position of the nodes in the image was computed by minimizing the ‘energy’ of the system. Moreover, the “analysis” section of STRING, gives some brief statistics of the inferred network, such as the number of nodes and edges, the average node degree and the clustering coefficient. Highly connected networks have high values. Moreover, also reported are the expected number and the protein–protein interaction (PPI) enrichment *p*-value that indicate that the nodes are not random and that the observed number of edges is significant. STRING analysis also consents to perform an enrichment analysis for Gene Ontologies that shows terms that were more enriched in the set of proteins in the network, regarding Biological Process, Molecular Functions, and Cellular Components. Interestingly, STRING suggests possible involved molecular pathways related to KEGG (Kyoto Encyclopedia of Genes and Genomes).

## 3. Results

### 3.1. Clinical Characteristics of Participants

A total of 40 follicular fluid samples were isolated from patients who underwent assisted reproduction. All patients included in this study were normovulatory and were divided into two main groups, according to patient age, a specific parameters affecting the female fertility; therefore, the group of young women (Y group) included women aged <34 years (average age 30.9 ± 1.7; *n* = 17), and old women (group O), women aged > 38 years (average age 41.1 ± 2.3; *n* = 23). When the enrolled patients were grouped according to the age, no significant differences were found in FSH, LH, or E2 levels on the day of hCG injection.

### 3.2. Analysis of the Redox State in the Follicular Fluid of Normovulatory Women According to Reproductive Age

Based on the knowledge of how critical the choice of different methodologies is, we decided to measure the non-enzymatic antioxidant capacity (NEAC) in FF of patients of both groups by means of two different approaches: the ferric reducing/antioxidant power (FRAP) and the trolox equivalent antioxidant capacity-AzinoBisThiazoline Sulfonic (TEAC-ABTS).

**Figure 1:**
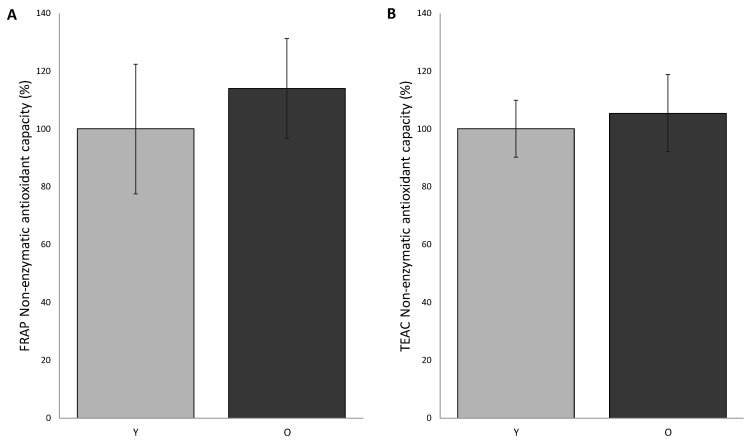
Total non-enzymatic antioxidant capacity of follicular fluids from young (Y) and old (O) women measured by means of ferric reducing/antioxidant power (FRAP) (**A**) and TEAC-ASBT (**B**). Values are expressed as % as respect to control (group Y), considered to be 100%. The values are the mean of three determinations ± SD. Biochemical analysis was carried out in duplicate in each biological sample.

The FRAP analysis was performed by comparing the average of the individual readings of the samples in O group with that of the Y group, and by setting the average value of the young group as 100%. According to this procedure, we observed that non-enzymatic antioxidant systems in FF of young and old patients are not significantly different (group Y 100.0 ± 22.5%; group O 114.26 ± 17.26%) (Figure 1).

The other approach (TEAC-ABST) confirmed this trend (100.0 ± 9.8% in young and 105.4 ± 13.3% in old women).

### 3.3. Comparative Analysis of the Total Free Thiol Groups Levels in the Follicular Fluid

**Figure 2:**
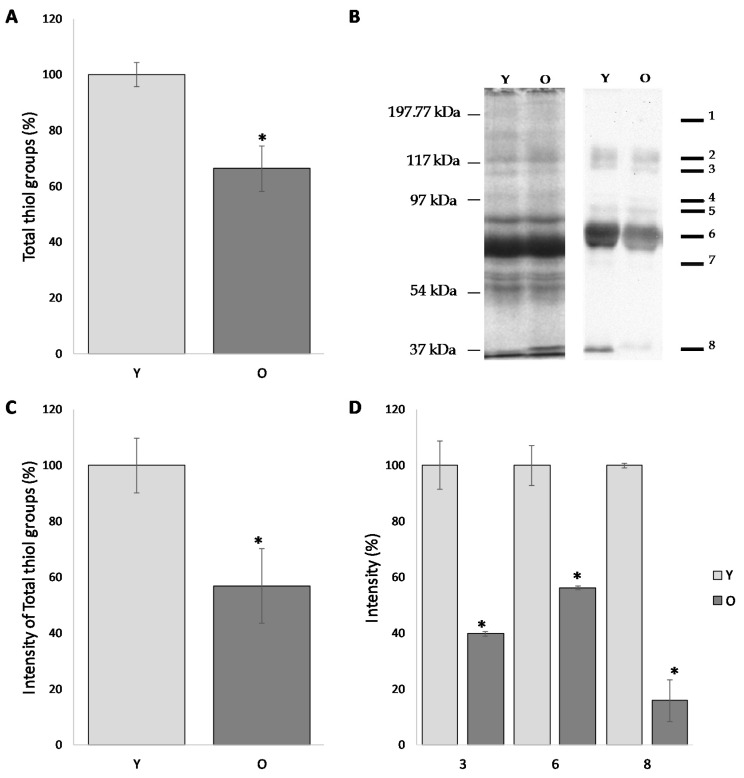
Total thiol groups in follicular fluids from young (Y) and old (O) women. (**A**) The level of total free –SH groups evaluated by colorimetric assay based on 5,5′-dithiobis- 2-nitrobenzoic acid (DTNB) staining. (**B**) Western blot analysis of proteins from FF, stained with silver nitrate (left) or MBP (right). The 8 predominant bands (molecular weight of 190, 120, 115, 90, 80, 70, 60, and 30 kDa) are indicated. (**C**) Graph of the intensity values (pixel intensity/mm^2^) detected by western blotting were normalized to the intensity values of the corresponding bands highlighted by the silver nitrate staining. (**D**) Relative intensities of bands 3-6-8 detected in both Y and O groups. The values were reported in percentage, considering the relative intensities of Y group as 100%. In this case the intensity of the individual bands of the spot (expressed as volume) was normalized on the intensity values of the same bands in the silver staining, in order to verify that the differences in the level of SH free observed were not due to the differential expression of the protein but of differences in the level of post-translational modification. * *p* < 0.05.

The level of total free -SH groups firstly evaluated by colorimetric assay revealed significant age-dependent differences. As showed in Figure 2A, the level of free -SH in the FF of young women is decreased of about 40% if compared to the older women (100.0 ± 4.2% and 63.3 ± 8.2%, respectively) (*p* < 0.05).

3-(N-maleimido-propionyl) biocytin, a biotin-containing, thiol-specific reagent, when used in combination with appropriate avidin-conjugated markers, represents a suitable and very sensitive thiol-specific probe, useful to detect protein SH groups on dot blots. To this end, once settled up the experimental conditions, proteins from FF have been stained with MBP, separated by SDS-PAGE and blotted on a nitrocellulose. The intensity values (pixel intensity/ mm^2^) detected by western blotting were normalized to the intensity values of the corresponding bands highlighted by the silver nitrate staining.

As shown in Figure 2B, follicular fluid proteins from both groups display an almost similar pattern, with 8 predominant bands characterized by a molecular weight of approximately: 190, 120, 115, 90, 80, 70, 60 and 30 kDa (Figure 2B). The semi-quantitative analysis of the total intensities was carried out by normalizing the intensity of group O to the group Y (considered as 100%). According to this procedure, the level of free -SH in the FF of old women was reduced by 43.2% compared to those of the young women (*p* < 0.05) (Figure 2C).

Interestingly, the comparative analysis of the intensities of the individual band provides evidence that the results obtained with the total intensity are confirmed for each individual band. In particular, when compared to the corresponding value in older women, bands 3, 6 and 8 are significantly decreased (39.8 ± 0.83%, 56.2 ± 0.66% and 15.9 ± 7.42%, respectively; *p* < 0.05) (Figure 2D).

This result supports the hypothesis that the proteins of the follicular fluids of older women showed reduced amounts of free thiol groups than young women; this datum is in line with the results from colorimetric assay.

### 3.4. Comparative Analysis of Free Thiol Groups by Two-Dimensional Electrophoresis and Western Blotting

In order to investigate the significant differences that emerged in the total amounts of free thiol groups, proteins from follicular fluids from both young and old women were analyzed by two-dimensional electrophoresis and western blotting, followed by qualitative and semiquantitative analysis of the spots.

**Figure 3:**
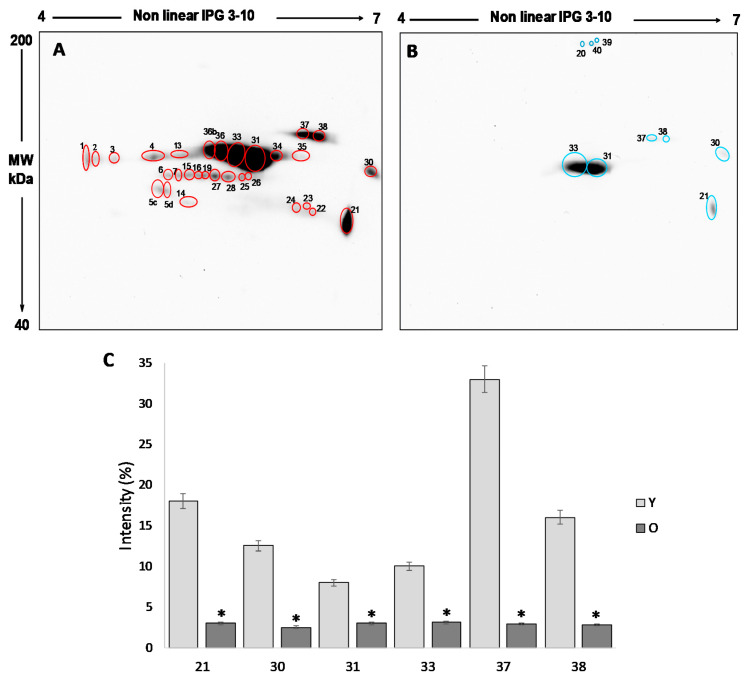
Avidin-blotting after 2D-electrophoresis of biotin-labelled free -SH residues in follicular fluid from young (panel **A**) and old (panel **B**) women. 2D electrophoresis was independently carried out in pooled samples in triplicate for each group, giving similar results. (**C**) The graph shows the six spots detected in both young and old women that are significantly more intense in group Y than in group O The values are the mean ± SD. * *p* < 0.05.

The analysis of the 2D blot of FF protein from group Y (Figure 3A) revealed 30 spots, distributed between pH 4 and pH 7 and with a molecular weight ranging from about 200 kDa to 60 kDa. In the 2D blot from O group, only 9 spots were displayed (Figure 3B): six already detected in the blot of young women and three (spot 20, 39 and 40) newly identified (Figure 3B).

For the semi-quantitative analysis, the intensity of the spots detected in the 2D blots of young women (considered as a control) was compared to those detected in old women. To this end, only spots having a mean intensity of 2.5-fold higher or lower than the corresponding spots in the control (*p* < 0.05) were considered. In order to confirm that any difference in the level of free-SH observed was due to differences in the level of post-translational modification, the intensity of each spot was normalized respect to the intensity of the corresponding spot in the silver stained gel.

This procedure leads us to demonstrate that the six spots detected in both young and old women are significantly more intense in group Y than in group O (*p* < 0.05) (Figure 3C), confirming a significant reduction in the free -SH groups of the FF proteins in older women.

By means of mass spectrometry, we identified some of the proteins displaying significant different levels of free -SH between the two groups (Table 1). In particular, among the co-expressed proteins having a higher level of free -SH in the FF of young women (more than 2.5-fold *p* < 0.05), we detected transferrin and albumin isoforms, while among the proteins detectable only in young women, we identified the isoforms of the heavy chains of immunoglobulin A and angiotensinogen.

### 3.5. Comparative Analysis of the Carbonylation Level in Follicular Fluid

As respect to the measurement of other oxidation products, the use of protein carbonyl groups to assess the extent of oxidative stress has some advantages because of the relative early formation and the relative stability of carbonylated proteins. Based on this knowledge, we detected carbonylated proteins in FF by means of two-dimensional high resolution electrophoresis together with methods for the detection of oxidized carbonylated proteins (derivatization with DNP and use of anti-DNP antibodies).

**Figure 4:**
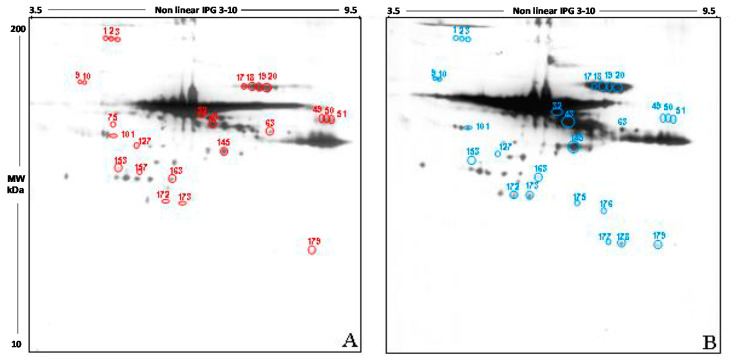
Western blotting after two dimensional electrophoresis showing carbonyl group in follicular fluid from young (**A**) and old (**B**) women. 2D electrophoresis was independently carried out in pooled samples in triplicate for each group, giving similar results.

The qualitative analysis carried out in the 2D blot of the Y group, chosen as control, showed 191 spots, of which 188 also detectable in the blot from the O group (Figure 4A). The 2D blot of old women revealed 192 spots, distributed between pH 3.5 and pH 9.5 and with a molecular weight ranging from 200 kDa to about 30 kDa (Figure 4B). Interestingly, 3 spots seem to be present only in FF proteins of young women (spots 75, 127, 157), while 4 spots are detectable only in old women (spots 175, 176, 177, and 178).

The semi-quantitative analysis of the total intensity was carried out by normalizing the intensity of O group to the Y group (considered as 100%). According to this procedure, the level of carbonyl groups in the follicular fluid of old women was increased by 30.5% compared to those of the young women (*p* < 0.05).

Also, in this case, only spots having a mean intensity of 2.5-fold higher or lower than the corresponding spots in the control blots were considered. In order to confirm that any difference in carbonylation observed was due to differences in the level of post-translational modification, the intensity of each spot was normalized as respect to the intensity of the corresponding spot in the silver stained gel.

This analysis showed that 21 spots display a significant difference in the intensity in the two study group, namely: spots 1, 2, 3, 9, 10, 17, 18, 19, 20, 32, 43, 49, 50, 51, 63, 101, 145, 153, 172, 273, 179. 14 spots have a higher intensity in FF proteins from young women if compared to the older (spots 9, 10, 17, 18, 19, 20, 32, 43, 63, 101, 145, 172, 273, 179); by contrast, 7 spots show a higher intensity in old women compared to the young (1, 2, 3, 49, 50, 51, 153).

### 3.6. STRING Analysis

**Figure 5:**
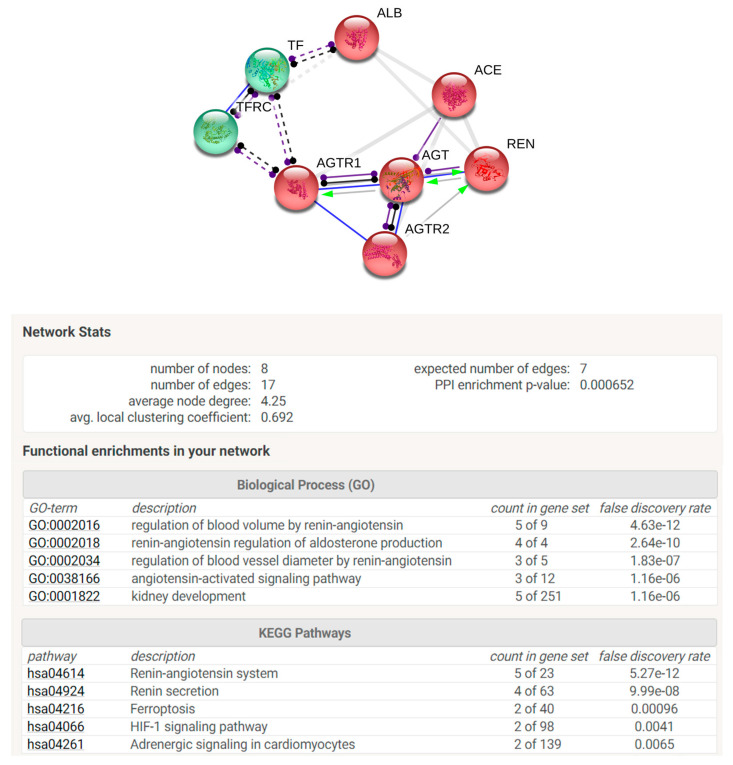
STRING analysis of the differentially abundant proteins reporting protein network with relative stats and functional enrichment by Biological Process and KEGG pathways.

STRING analysis was performed in order to elaborate an enrichment analysis, summarizing functional information about the identified proteins with respect to the biological process Gene Ontology category and to predict possible associations among them. STRING networks consist of nodes (spheres) representing proteins, while edges represent the predicted mode of action among proteins (Figure 5). The analysis was elaborated in order to connect our protein species with a maximum of 5 more interactors. In the network are reported identified proteins connected with the predicted functional partners such as TFRC (transferrin receptor protein 1), AGTR1 (type 1-angiotensin II receptor), ACE (angiotensin-converting enzyme), REN (renin), AGTR2 (type 2-angiotensin II receptor). The two colours of the spheres (green and red) identify the 2 clusters of functionally correlated proteins. Proteins reported in the net are involved in regulation of blood volume by renin–angiotensin system and in renin–angiotensin regulation of aldosterone production biological processes. Moreover, among KEGG pathways are highlighted renin–angiotensin system, renin secretion, ferroptosis, Hypoxia-inducible factor (HIF-1) signalling pathway, and adrenergic signalling.

## 4. Discussion

In recent years, an ever-increasing interest of the scientific community has been directed to the study of the pathophysiological role of radical species. From both a scientific and clinical point of view, the analysis of the effects of oxidative stress on female fertility represents a topic of great interest [6,24]. A great deal of research has shown a contributory role of follicular fluid ROS on ovarian aging and consequently, on oocyte quality [25,26,27].

By means of FRAP and TEAC, we demonstrate that the non-enzymatic antioxidant capacity in FF of young and old women are comparable, highlighting, at the same time, a high inter-individual variability. This observation seems to be not in agreement with results from Carbone et al., demonstrating that FF of older women exhibited a lower level of enzymatic antioxidant defenses [28]. Anyway, we would like to point out that unlike enzymatic activity, the concentration of antioxidant molecules, such as vitamins, is closely related to the individual lifestyles and eating habits, as confirmed by the high inter-individual variability we detected in our cohort. Moreover, we would like to point to the significant limitations of most of the techniques so far used to quantify oxidative stress [29,30]; therefore, the setup of a suitable methodology to properly measure antioxidant capacity and the identification of molecular targets is advisable.

The combined approach we used in this study represents an interesting option, based on the observation that ROS are generated and dissolve rapidly but they induce stable modifications at the protein level (identified as oxidative damage to the proteins), which can lead to the proteolytic breakdown of the peptide bond, crosslinking, and/or modifications at the level of the side chains of amino acids such as carbonylation or the formation of bridges intramolecular and intermolecular disulphide. These modifications may change the protein function and their antigenicity, therefore potential inducing immunological processes associated with the inflammatory response and/or autoimmune damage. This impaired regulation of the immune system activation, along with the unbalanced production of ROS may result in an oxidative damage. To the best of our knowledge, the synergic action of oxidative stress and immunity has been reported to cause many pathological conditions affecting the female fertility [31]. Therefore, the evaluation of the carbonylation level of the proteins and the level of free -SH were used in this study as markers of the oxidation level in the follicular fluid.

Our innovative approach based on the use of 2D-PAGE/Western Blotting coupled with the staining of free thiol groups with a biotinylated derivative of maleimide and of carbonyl groups with dinitrophenylhydrazine, demonstrated that both the oxidation of the -SH and the carbonylation of the proteins, induced by oxidative stress, occurs in a specific and selective way.

The free protein thiols evaluation showed a more marked difference between the oxidative damage in young and old women; the relative semi-quantitative analysis on the total free -SH level confirmed a significant reduction in old women when compared to the younger. The effectiveness of our strategy is in agreement with previous studies showing that micronutrients supplementation before IVF cycles has a positive effect on follicular milieu by increasing the total non-enzymatic antioxidant capacity and by decreasing the oxidative damage on FF proteins [8] and that Metformin administration recovered a significantly high level of free -SH groups in FF proteins of polycystic ovarian syndrome (PCOS) women clearly indicating a decrease of OS level with respect to that found in FF samples from control [9].

By means of mass spectrometry, we identified some proteins that resulted differently stained between the two categories. In particular, some isoforms of albumin and transferrin were more than 2.5-fold more abundant in FF of young women compared to older women. With regard to the albumin isoforms, it has been reported that serum albumin acts as an antioxidant in human body fluids [32]; indeed, multiple oxidation sites have been identified in this protein. Moreover, our data are in agreement with the decreased level of the reduced form of human albumin detected in serum of elderly [33], thus confirming that some structural alterations in circulating albumin molecules accumulate with age. Another important function of serum albumin may be to participate in the maintenance of a constant redox potential in the extracellular fluids, thus securing a certain redox buffer capacity. Indeed, the relative abundance of the reduced form of albumin might reflect this variation in the redox buffer capacity with aging. However, the exact structural modifications or alterations of albumin related to the aging are not fully understood.

Another important finding of this study is the identification, by mass spectrometry, of some isoforms of the α chains of the immunoglobulins and the angiotensinogen that are specifically detected only in follicular fluid of young women. In this context, it would be particularly interesting to clarify the possible role of oxidation of some angiotensinogen isoforms. Angiotensinogen is a glycoprotein belonging to the class of α2-globulins, from which angiotensin I and angiotensin II are produced by renin enzyme. The RAS system (Renin–Angiotensin System) suggested by enrichment analysis which regulates blood pressure and electrolyte metabolism, seems to play an important role in the ovarian follicle by regulating steroidogenesis, oocyte maturation and follicle atresia. By both the autocrine and paracrine way, the RAS system affects follicular neovascularization [34]. Moreover, it seems to be involved in PCOS, ovarian hyperstimulation syndrome, ovarian tumors, and ectopic pregnancies [35]. The correlation between the increase in the oxidative state and reproductive aging has not yet been clarified, although data in the literature suggest the reduction of vascularization due to vascular atherosclerosis of the follicle may be a possible cause [36]. Interestingly, among KEGG pathways is reported HIF-1 signalling pathway, largely reported to activate the hypoxia response elements (HREs) following oxidative stress [37]

The carbonylation of proteins is instead an irreversible more complex process, playing an important physiologic role in the protein quality control, since the carbonylated proteins are more susceptible to proteolytic degradation [38]. Protein carbonylation is, therefore, a marker of a serious oxidative damage, occurring, for example, in pathological conditions such as cancer or age-associated diseases such as Alzheimer’s and Parkinson’s [39,40], but also in the oocyte aging [41]. Interesting insights come from the analysis of spots differentially detected in the two groups. The first intriguing observation is that all the proteins showing decreased free -SH group in old women, are also highly carbonylated, such as transferrin and angiotensinogen. Moreover, the highly carbonylated isoelectric series we detected in proteins from FF of both young and old women are represented by albumin, already known for its scavenging activity, and by the heavy chains of the G immunoglobulins. Since both albumin and immunoglobulins are most represented proteins in biological fluids, it may be hypothesized that their abundance may affects the probability that these proteins can be oxidized. Anyway, we detected that several spots are strongly stained in immunoblotting while they are barely detectable in the silver-stained gel. Therefore, this hypothesis not appears to be true and other factors seems to be involved. To this regard, from the literature, we know that the sensitivity of proteins to oxidation can be influenced by the structure of the protein itself. It is known that oxidation of carbohydrates in glycoproteins can contribute precisely to the incorporation of carbonyls (glycoxidation reaction) [42]. The presence of metal-binding sites on proteins also increases the possibility of these proteins experiencing oxidation. The carbonyl formation reaction is in fact catalyzed by positive ions and in particular by iron: the binding of Fe^2+^ to a specific site on the protein is followed by the reaction with peroxides during which reactive species are generated. These react mainly with amino acids present in the binding site for metals [42]. Transferrin, for example, contains eight ion binding sites for iron, while albumin contains one binding site for the copper ion.

## 5. Conclusions

In conclusion, even if a great deal of research is needed to shed light on the contributory role of oxidative modifications in ovarian aging, we provide evidence for a different pattern of oxidative damage in follicular fluid of young as respect to old women, despite comparable levels of non-enzymatic antioxidant defense. Importantly, we definitively demonstrated that the level of free -SH, along with the level of carbonylated group, represents a powerful index of both the protein’s oxidative damage and of the residual buffering capacity of the system under analysis.

These insights pave the way to further studies aiming to shed light on the molecular mechanisms underlining the OS in the follicular microenvironment in conditions of sub-fertility, associated with a reduction in the reproductive capacity linked to the patient’s aging.

## Figures and Tables

**Table 1:** List of proteins identified in follicular fluid differently expressed in young patients than in old. The table reports the protein name, the accession number, theoretical and experimental pI and molecular weight (MW). Mascot Search Results comprehending number of matched peptides, sequence coverage and score.

ID	Protein Name	Entry Name	AC	Theoretical pI/Mr (kDa)	Experimental pI/Mr (kDa)	Mascot Search Results		
						No. of Matched Peptides	Sequence Coverage (%)	Score
5c ^#^	Angiotensinogen	ANTG	P01019	5.87/53.40	5.08/62.60	13	27	130
5d ^#^	Angiotensinogen	ANTG	P01019	5.87/53.40	5.10/62.58	10	20	106
6 ^#^	Immunoglobulin heavy chain α	IGHA	P01876	6.08/38.49	5.31/62.61	5	14	65
7 ^#^	Immunoglobulin heavy chain α	IGHA	P01876	6.08/38.49	5.38/62.59	5	14	68
15 ^#^	Immunoglobulin heavy chain α	IGHA	P01876	6.08/38.49	5.51/62.60	7	16	80
16 ^#^	Immunoglobulin heavy chain α	IGHA	P01876	6.08/38.49	5.60/62.59	5	12	53
19 ^#^	Immunoglobulin heavy chain α	IGHA	P01876	6.08/38.49	5.65/62.61	6	16	83
27 ^#^	Immunoglobulin heavy chain α	IGHA	P01876	6.08/38.49	5.69/62.61	8	18	94
28 ^#^	Immunoglobulin heavy chain α	IGHA	P01876	6.08/38.49	5.72/62.61	7	21	91
37 *	Transferrin	TRFE	P02787	6.81/79.28	6.35/80.80	21	31	212
38 *	Transferrin	TRFE	P02787	6.81/79.28	6.41/80.78	26	35	248
31 *	Serum albumin	ALBU	P02768	5.92/71.31	5.84/80.61	21	33	245
33 *	Serum albumin	ALBU	P02768	5.92/71.31	5.75/80.65	19	38	215
33b *	Serum albumin	ALBU	P02768	5.92/71.31	5.68/80.64	22	34	232
34 *	Serum albumin	ALBU	P02768	5.92/71.31	5.90/80.64	19	35	225
36 *	Serum albumin	ALBU	P02768	5.92/71.31	5.64/80.66	21	39	252
36b *	Serum albumin	ALBU	P02768	5.92/71.31	5.61/80.65	18	32	212

* Protein with a mean intensity of 2.5-fold higher in Y group than in the O group (*p* < 0.05). ^#^ detected only in FF from Y group.
